# Citreoviridin Induces Autophagy-Dependent Apoptosis through Lysosomal-Mitochondrial Axis in Human Liver HepG2 Cells

**DOI:** 10.3390/toxins7083030

**Published:** 2015-08-06

**Authors:** Yuexia Wang, Yanan Liu, Xiaofang Liu, Liping Jiang, Guang Yang, Xiance Sun, Chengyan Geng, Qiujuan Li, Xiaofeng Yao, Min Chen

**Affiliations:** 1 Department of Preventive Medicine, Dalian Medical University, 9 W Lvshun South Road, Dalian 116044, China; E-Mails: wangyuexia1990@hotmail.com (Y.W.); liuyanan_sh@hotmail.com (Y.L.); food@dlmedu.edu.cn (X.L.); jianglipingdl@163.com (L.J.); yg290@163.com (G.Y.); sunxiance@gmail.com (X.S.); gengchengyandl@gmail.com (C.G.); liqiujuandl@gmail.com (Q.L.); 2 Liaoning Anti-Degenerative Diseases Natural Products Engineering Research Center, Dalian Medical University, 9 W Lvshun South Road, Dalian 116044, China

**Keywords:** citreoviridin, autophagy, apoptosis, lysosomal membrane permeabilization, human liver HepG2 cells

## Abstract

Citreoviridin (CIT) is a mycotoxin derived from fungal species in moldy cereals. In our previous study, we reported that CIT stimulated autophagosome formation in human liver HepG2 cells. Here, we aimed to explore the relationship of autophagy with lysosomal membrane permeabilization and apoptosis in CIT-treated cells. Our data showed that CIT increased the expression of LC3-II, an autophagosome biomarker, from the early stage of treatment (6 h). After treatment with CIT for 12 h, lysosomal membrane permeabilization occurred, followed by the release of cathepsin D in HepG2 cells. Inhibition of autophagosome formation with siRNA against Atg5 attenuated CIT-induced lysosomal membrane permeabilization. In addition, CIT induced collapse of mitochondrial transmembrane potential as assessed by JC-1 staining. Furthermore, caspase-3 activity assay showed that CIT induced apoptosis in HepG2 cells. Inhibition of autophagosome formation attenuated CIT-induced apoptosis, indicating that CIT-induced apoptosis was autophagy-dependent. Cathepsin D inhibitor, pepstatin A, relieved CIT-induced apoptosis as well, suggesting the involvement of the lysosomal-mitochondrial axis in CIT-induced apoptosis. Taken together, our data demonstrated that CIT induced autophagy-dependent apoptosis through the lysosomal-mitochondrial axis in HepG2 cells. The study thus provides essential mechanistic insight, and suggests clues for the effective management and treatment of CIT-related diseases.

## 1. Introduction

Citreoviridin (CIT) is a toxic secondary metabolite derived from *Penicillum citreonigrum*, *Aspergillus terreus* and *Eupenicillium ochrosalmoneum* in moldy cereals, such as rice and corn [1]. It has been shown that the consumption of CIT-contaminated yellow rice is associated with the occurrence of cardiac beriberi in Japan, and Keshan disease in China and South East Asian countries [2]. CIT interferes with nerve and muscle tissues metabolism by competitively inhibiting the absorption of vitamin B_1_, thus causing beriberi [3]. Following 10 days of subcutaneous CIT injection, liver phosphatases were significantly decreased and glutamic oxaloacetic transaminase was increased in rats [4]. Compared with its neurotoxicity and cardiotoxicity, CIT hepatotoxicity has been less investigated and remains largely unknown.

In our previous study, we reported that CIT stimulated autophagosome formation and caused autophagic cell death in HepG2 cells [5]. Autophagy and apoptosis regulate the turnover of organelles and proteins within cells. In general, autophagy blocks the induction of apoptosis, while apoptosis-associated caspase activation shuts off the autophagic process. However, in special cases, autophagy or autophagy-relevant proteins may induce apoptosis [6]. Autophagy and apoptosis can occur in the same cell, mostly in a sequence in which autophagy precedes apoptosis [7]. This observation aroused our interest in investigating the incidence of apoptosis and autophagy, and any possible relationship that may exist, in CIT-treated cells.

Many signal transduction pathways elicited by cellular stress regulate both autophagy and apoptosis [6]. The cytosolic pool of p53 represses autophagy and the nuclear translocation of p53 facilitates the induction of autophagy [8]. In conditions of cellular stress, a portion of cytosolic p53 can translocate to the mitochondrial matrix, where p53 promotes opening of the permeability transition pore (PTP) [9]. PTP opening is one of the mechanisms causing mitochondrial outer membrane permeabilization (MOMP), thereby setting off the apoptotic cascade [10]. Several BCL-2 homology 3 (BH3)—only proteins have the dual capacity to activate both autophagy and apoptosis. Ser/Thr kinases, including JUN *N*-terminal kinase (JNK), death-associated protein kinase (DAPK) and protein kinase B (AKT), have also been implicated in the regulation of both autophagy and apoptosis [6]. An autophagy-dependent mechanism has been demonstrated in the activation of caspase-8 and initiation of the apoptotic cascade in response to SKI-I, a pan-sphingosine kinase inhibitor, and bortezomib, a proteasome inhibitor [11]. Autophagy-related gene (Atg) 7 facilitates the induction of apoptosis after lysosomal photodamage, presumably by triggering lysosomal membrane permeabilization (LMP) [12]. Lysosomal release of cathepsins can also trigger MOMP, thereby stimulating the apoptotic pathway [13].

In this study, we aimed to investigate the effects of CIT treatment on LMP and the role of LMP in CIT-induced apoptosis. We also examined the relationship between autophagy and apoptosis in CIT-treated cells.

## 2. Results 

### 2.1. CIT Increased the Expression of LC3-II from the Early Stage of Treatment

**Figure 1:**
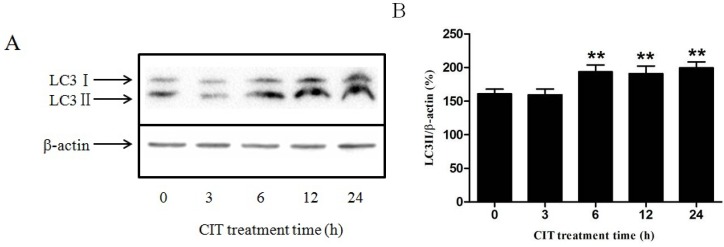
The expression of LC3-II in HepG2 cells after treatment with CIT. HepG2 cells were treated with 5 µM CIT for 3 h, 6 h, 12 h, or 24 h. (**A**) The cytoplasmic protein fraction was analyzed by Western blot. -actin was used as an internal control. (**B**) Densitometric analyses of LC3-II expressed in HepG2 cells. The relative expression of LC3-II was expressed as a percentage of the level of -actin (*n* = 3). The bar represents mean ± SD. ** *p* < 0.01 *vs.* control.

Microtubule-associated protein light chain 3 (LC3)-II is considered the most reliable biochemical marker of autophagy [14]. Our data showed that the expression of LC3-II was significantly increased after treatment with 5 M CIT for 6 h, 12 h, and 24 h. After treatment with 5 µM CIT for 3 h, the LC3-II levels did not change significantly as compared with the control (Figure 1). 

### 2.2. CIT-Induced LMP Was Autophagy Dependent

**Figure 2:**
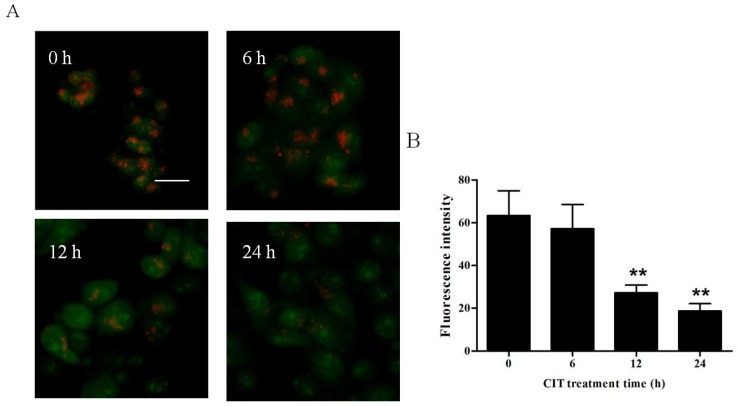
CIT induced lysosomal membrane permeabilization in HepG2 cells. HepG2 cells were treated with 5 µM CIT for 6 h, 12 h, or 24 h. (**A**) Lysosomal membrane stability in CIT-treated HepG2 cells was measured by AO staining under a fluorescence microscope (scale bar: 20 µm). (**B**) Quantification of red fluorescence intensity of AO in HepG2 cells. The bar represents the means ± SD (*n* = 6). ** *p* < 0.01 *vs.* control.

**Figure 3:**
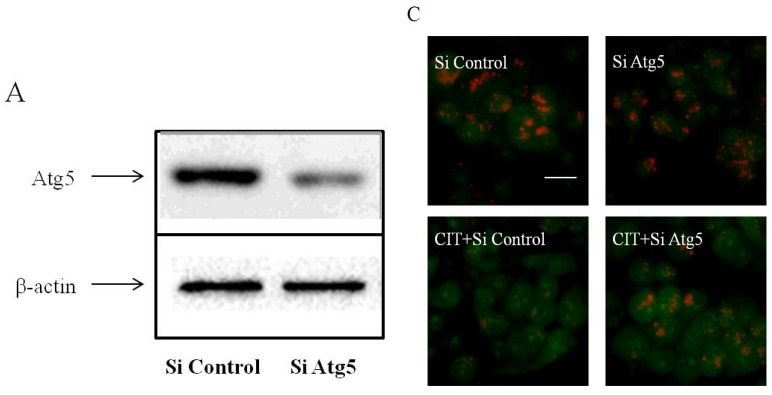

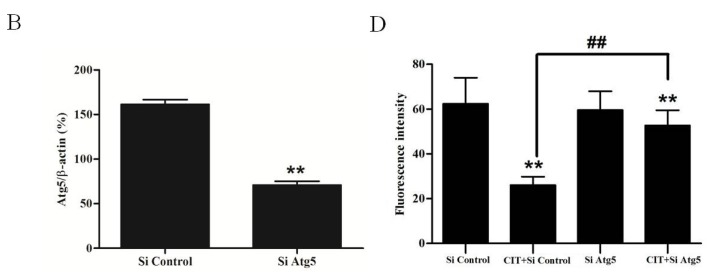
Effect of siRNA against Atg5 on CIT-induced LMP in HepG2 cells. HepG2 cells were transfected with either 50 nM siRNA against human Atg5 (Si Atg5) or scrambled control siRNA (Si Control), and then treated with 5 µM CIT for 12 h. (**A**) SiRNA transfection efficiency was assessed by Western blot after 48 h of transfection. -actin was used as an internal control. (**B**) Densitometric analyses of Atg5 expressed in HepG2 cells. Relative expression of Atg5 was expressed as a percentage of the level of -actin. (**C**) Lysosomal membrane stability in CIT-treated HepG2 cells was measured by AO staining under a fluorescence microscope (scale bar: 20 µm). (**D**) Quantification of red fluorescence intensity of AO in HepG2 cells. The bar represents mean ± SD (*n* = 6). ** *p* < 0.01 *vs.* control; ^##^ *p* < 0.01.

We performed acridine orange (AO) staining to examine lysosomal membrane stability. As shown in Figure 2, after treatment with 5 µM CIT for 12 h and 24 h, the numbers of red puncta (intact lysosome) were significantly decreased as compared with the control. This suggests CIT to have caused significant LMP in HepG2 cells. After treatment with 5 µM CIT for 6 h, the intensity of red fluorescence did not change significantly as compared with the control. Taken together with the evidence of early increased expression of LC3-II, these data suggested that CIT-activated autophagosome formation preceded LMP. To evaluate the role of autophagy in CIT-induced LMP, HepG2 cells were pretreated with siRNA against Atg5 before treatment with CIT. Knockdown of Atg5 attenuated CIT-induced LMP in HepG2 cells (Figure 3). These data indicated that CIT-induced LMP was autophagy dependent.

### 2.3. Inhibition of Autophagosome Formation Alleviated CIT-induced Lysosomal Release of Cathepsin D

**Figure 4:**
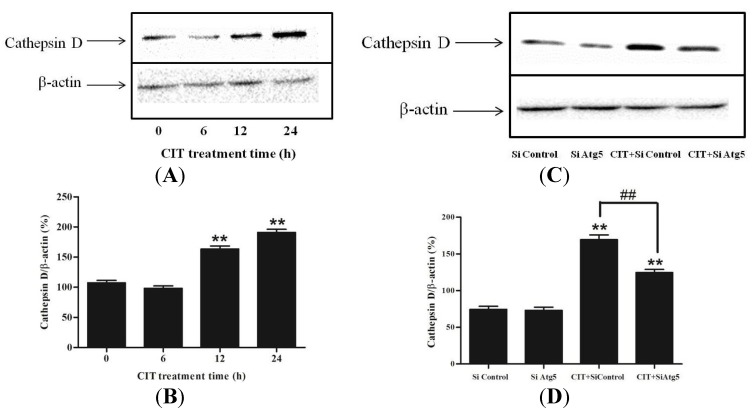
Inhibition of autophagosome formation attenuated CIT-induced lysosomal release of cathepsin D in HepG2 cells. The cytosolic extracts were analyzed by Western blot. -actin was used as an internal control. The relative level of cathepsin D was expressed as a percentage of the level of -actin (*n* = 3). (**A**) HepG2 cells were treated with 5 µM CIT for 6 h, 12 h, or 24 h. (**B**) Densitometric analyses of cytosolic cathepsin D levels in HepG2 cells as described in A. (**C**) HepG2 cells were transfected with 50 nM siRNA against human Atg5 (Si Atg5) or scrambled control siRNA (Si Control), and then treated with 5 µM CIT for 12 h. (**D**) Densitometric analyses of cytosolic cathepsin D levels in HepG2 cells as described in C. ** *p* < 0.01 *vs.* control; ^##^ *p* < 0.01.

Lysosomal release of cathepsin D is a causative event, leading to apoptosis [15]. After treatment with 5 µM CIT for 12 h and 24 h, cathepsin D levels in the cytosolic extracts were significantly increased as compared with the control (Figure 4A,B). After treatment with 5 µM CIT for 6 h, the cytosolic cathepsin D levels did not change significantly compared with the control. These results were consistent with the data of CIT-induced LMP. Additionally, knocking down Atg5 attenuated CIT-induced lysosomal release of cathepsin D in HepG2 cells (Figure 4C,D).

### 2.4. CIT-induced Collapse of Mitochondrial Transmembrane Potential (ΔΨm) Was Autophagy Dependent

**Figure 5:**
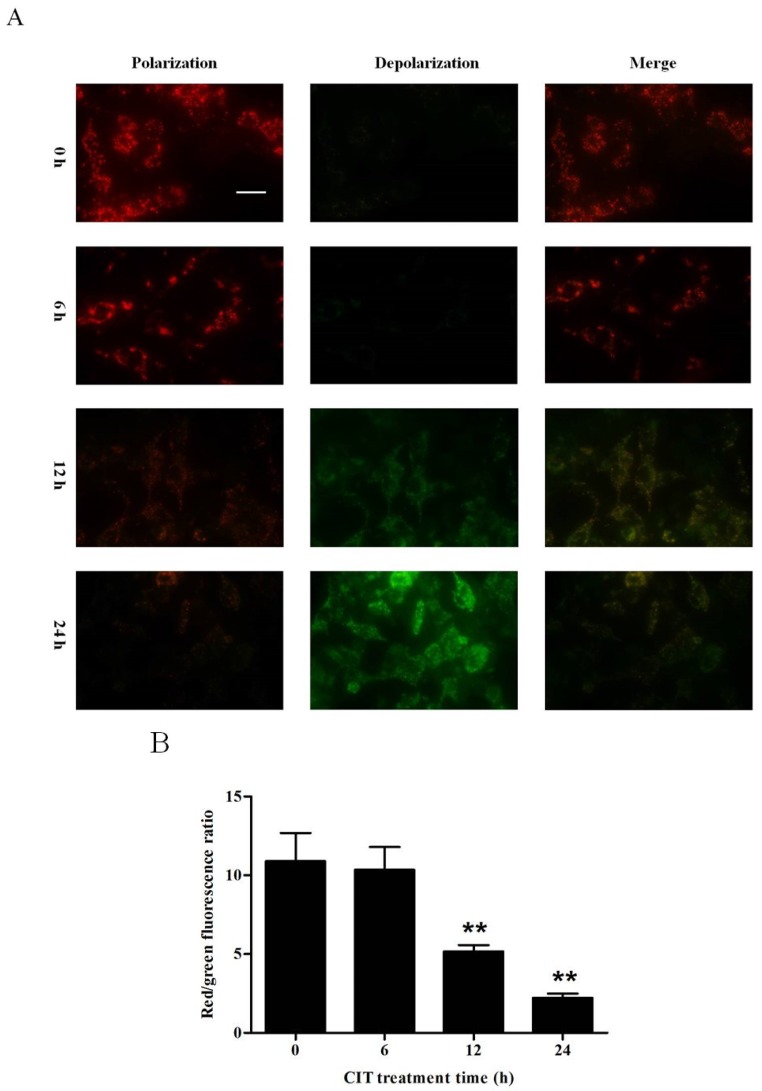

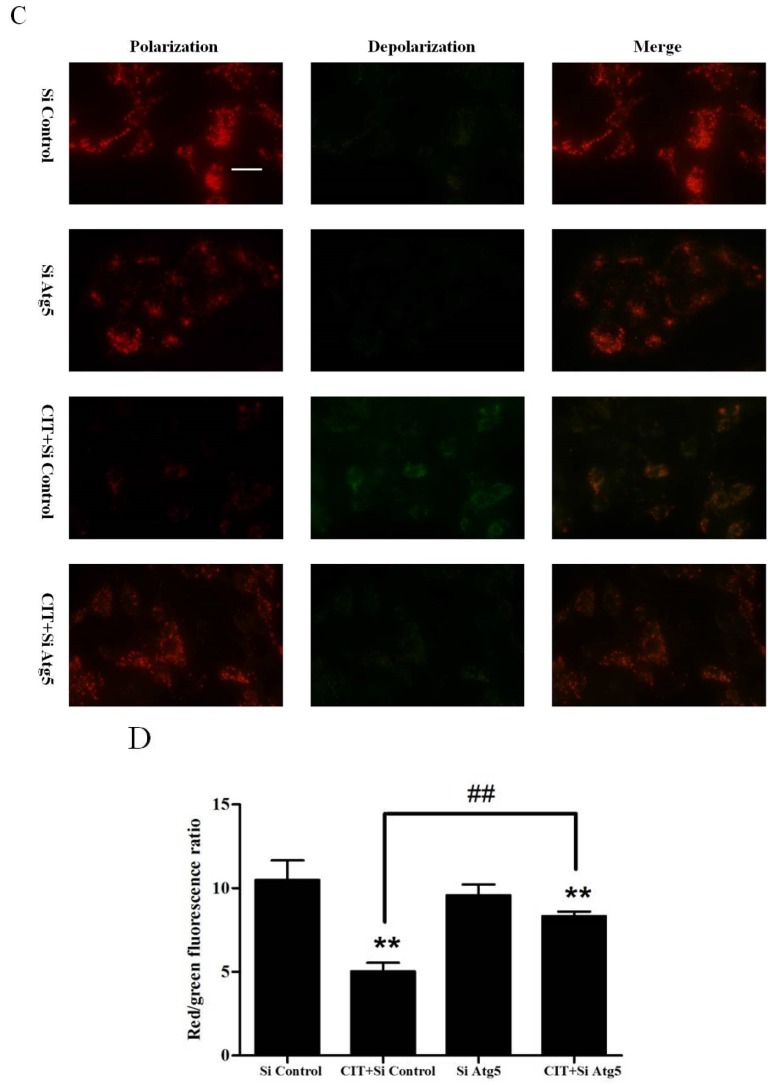
CIT-induced collapse of ΔΨm was autophagy dependent. ΔΨm of CIT-treated HepG2 cells was evaluated by JC-1 staining under a fluorescence microscope (scale bar: 20 µm). ΔΨm was represented by the ratio of JC-1 red fluorescence (aggregated form: indicating polarized/normal ΔΨm) to green fluorescence (monomeric form: indicating depolarized/low ΔΨm), *n* = 3. (**A**) HepG2 cells were treated with 5 µM CIT for 6 h, 12 h, or 24 h. (**B**) The ratio of red/green fluorescence in cells stained with JC-1 as described in A. (**C**) HepG2 cells were transfected with either 50 nM siRNA against human Atg5 (Si Atg5) or scrambled control siRNA (Si Control), and then treated with 5 µM CIT for 12 h. (**D**) The ratio of red/green fluorescence in cells stained with JC-1 as described in C. ** *p* < 0.01 *vs.* control; ^##^ *p* < 0.01.

Collapse of ΔΨm is a down-stream event of lysosomal cathepsin D release and a triggering event of apoptosis [16]. We performed JC-1 staining to examine the mitochondrial transmembrane potential of HepG2 cells. Upon treatment of HepG2 cells with 5 µM CIT for 12 h and 24 h, the ΔΨm of the cells was significantly deceased as compared with the control (Figure 5A,B). Knocking down Atg5 attenuated CIT-induced collapse of ΔΨm in HepG2 cells (Figure 5C,D).

### 2.5. CIT Induced Autophagy-Dependent Apoptosis in HepG2 Cells

**Figure 6:**
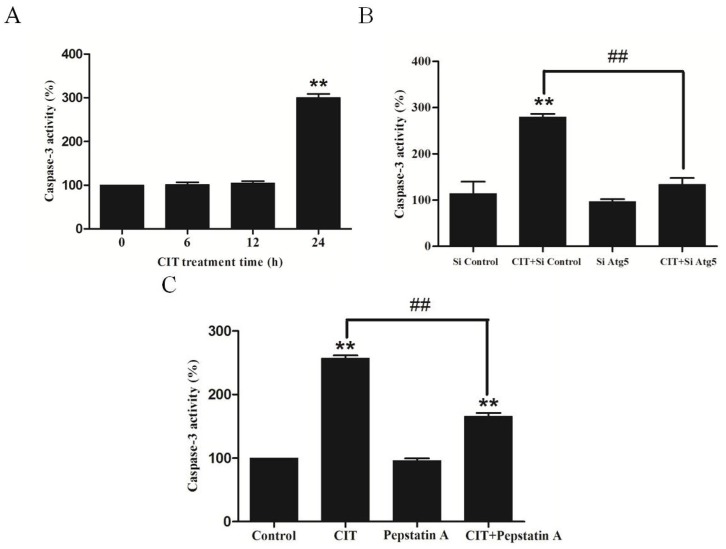
CIT induced autophagy-dependent apoptosis in HepG2 cells. Caspase-3 activity was determined using colorimetric method and was expressed as a percent of control activity. (**A**) HepG2 cells were treated with 5 µM CIT for 6 h, 12 h, or 24 h. (**B**) HepG2 cells were transfected with either 50 nM siRNA against human Atg5 (Si Atg5) or scrambled control siRNA (Si Control), and then treated with 5 µM CIT for 24 h. (**C**) HepG2 cells were pretreated with 40 µM pepstatin A for 4 h, and subsequently treated with 5 µM CIT for 24 h. ** *p* < 0.01 *vs.* control; ^##^ *p* < 0.01, *n* = 3.

We performed Caspase-3 activity analysis to examine cellular apoptosis. After treatment with 5 µM CIT for 24 h, apoptosis was induced in the cells (Figure 6A). The pretreatment of HepG2 cells with siRNA against Atg5 relieved CIT-induced apoptosis (Figure 6B), indicating that CIT-induced apoptosis was autophagy dependent. To evaluate the role of cathepsin D in CIT-induced apoptosis, HepG2 cells were pretreated with the cathepsin D inhibitor, pepstatin A, before treatment with CIT. The data showed that pepstatin A was able to relieve CIT-induced apoptosis (Figure 6C). These data indicated that the lysosomal-mitochondrial axis might play an important role in CIT-induced apoptosis.

## 3. Discussion

In our previous study, we found that CIT increased the autophagosome number in HepG2 cells, which was observed under a transmission electron microscope. Reduction of P62 protein levels and the result of LC3 turnover assay indicated that the accumulation of autophagosomes was due to increased formation rather than impaired degradation in HepG2 cells treated with 5 µM CIT for 24 h [5]. In this study, the formation of autophagosomes was activated considering the increase of LC3-II levels in HepG2 cells treated with 5 µM CIT for 6 h. Since activated autophagosome formation was an early event, it aroused our interest in investigating the relationship of autophagy with LMP and apoptosis in CIT-treated HepG2 cells.

In an effort to determine the role of autophagy in CIT-induced LMP and apoptosis, we pretreated cells with siRNA against Atg5. Atg5 is an essential component for the initiation of autophagy and the loss of Atg5 completely blocks the entire autophagy process [17]. 

CIT caused LMP in HepG2 cells after treatment with 5 µM CIT for 12 h, but not 6 h. This suggests that LMP may have occurred after autophagic flux activation in CIT-treated HepG2 cells. In addition, knocking down Atg5 alleviated CIT-induced LMP. These findings suggested that the activation of autophagic flux by CIT treatment could lead to LMP in HepG2 cells. It has also been reported that inhibition of autophagic response by wortmannin or asparagine reduced LMP in resveratrol-treated cervical cancer cells [18]. Autophagy is a highly conserved cellular process in which cytoplasmic materials, including organelles, are sequestered into double-membrane vesicles called autophagosomes and delivered to lysosomes for degradation or recycling [19]. The induction of autophagy may cause an increase in intralysosomal degradation of macromolecules or organelles, stimulation of intralysosomal redox-active iron, and the accumulation of oxidative lipoprotein. The accumulation of oxidized lipoproteins within lysosomes may negatively affect the integrity of lysosomal membranes and provide a stimulus for the induction of LMP [20]. 

The increased levels of cathepsin D in cytosolic extracts confirmed the CIT-induced LMP in HepG2 cells. Elevated levels of cytosolic cathepsin D were detected after treatment with 5 µM CIT for 12 h in the cells. Inhibition of autophagosome formation with siRNA against Atg5 attenuated CIT-induced lysosomal release of cathepsin D. Cathepsin D is an important mediator of the lysosomal-mitochondrial axis causing apoptosis. Upon LMP, the released cathepsin D induces conformational change of Bax and its translocation to the mitochondria, subsequently promoting MOMP [21]. In this study, MOMP was induced in CIT-treated HepG2 cells as indicated by the loss of ΔΨm. As anticipated, knocking down Atg5 also attenuated the CIT-induced MOMP in HepG2 cells.

**Figure 7:**
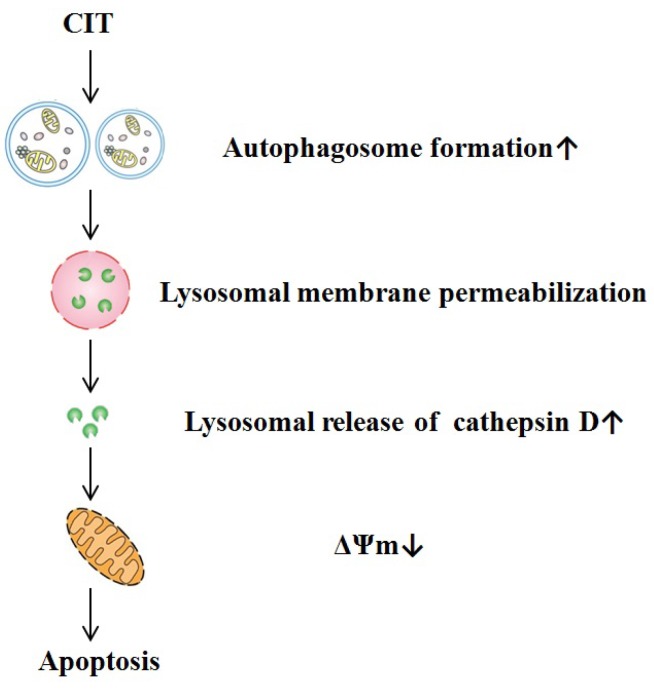
The proposed pathway of CIT-induced autophagy-dependent apoptosis in HepG2 cells. CIT-activated autophagic flux is an upstream event that may trigger LMP. CIT-induced LMP causes lysosomal release of cathepsin D and induces MOMP to eventually trigger mitochondrial apoptosis.

MOMP plays an essential role in mitochondrial apoptosis through releasing cytochrome c and other apoptogenic proteins into the cytosol [22]. CIT induced apoptosis in HepG2 cells after treatment for 24 h. We found that inhibition of autophagosome formation attenuated the CIT-induced apoptosis. These data indicated that the CIT-induced apoptosis could be autophagy-dependent. It has also been reported that 2-methoxyestradiol induced autophagy-dependent apoptosis in Ewing sarcoma cells, and the survivin suppressant, YM155, induced autophagy-dependent apoptosis in prostate cancer cells [23,24]. We found that cathepsin D inhibitor, pepstatin A [25], efficiently attenuated CIT-induced apoptosis. One plausible explanation for the observed CIT-induced autophagy-dependent apoptosis in HepG2 cells is that it is the ultimate outcome of the progressive effect or activity of CIT, that is, an early CIT-activated autophagic flux and a relatively later LMP in the cells. CIT-induced LMP causes lysosomal release of cathepsin D and induces MOMP, eventually triggering mitochondrial apoptosis (Figure 7).

Although autophagy is regarded as a normal degradative process maintaining cellular homeostasis, this process represents one mode of programmed cell death. Excessive levels of autophagy have been observed in association with various forms of cell death and the term “autophagic cell death” was introduced by the Nomenclature Committee on Cell Death (NCCD) to describe cell death that is suppressed by inhibition of the autophagy pathway [26]. The cell death induced by certain bacterial or vegetal toxins is reported to be autophagy dependent. In our previous study, we reported that CIT induced reactive oxygen species-mediated autophagic cell death in HepG2 cells [5]. Shiga toxins, virulence factors produced by the bacteria *Shigella dysenteriae* and certain strains of *Escherichia coli*, induce autophagic cell death in intestinal epithelial cells via the endoplasmic reticulum stress pathway [27]. Fumonisin B1, a mycotoxin produced primarily by *Fusarium verticillioides* and *Fusarium proliferatum*, induces autophagic cell death mediated by endoplasmic reticulum stress in monkey kidney MARC-145 cells [28]. 

In summary, we reported that CIT-activated autophagic flux might be an upstream event that triggered apoptosis through the lysosomal-mitochondrial axis. This study provides insight into further understanding the molecular mechanisms underlying CIT hepatotoxicity, and helps us improve the management and/ or treatment of CIT-related diseases.

## 4. Experimental Section 

### 4.1. Cell Culture and Treatment

Human hepatoma cell line HepG2 was purchased from American Type Culture Collection. HepG2 cells were cultured in MEM/EBSS medium (Hyclone, Logan, UT, USA) supplemented with 10% fetal bovine serum (Biological Industries, Kibbutz Beit Haemek, Israel) and antibiotics (100 U/mL penicillin and 100 g/mL streptomycin; Sigma, St. Louis, MO, USA) under humidified atmosphere with 5% CO_2_ at 37 °C. CIT (CAS No. 25425-12-1, Enzo Life Sciences, Farmingdale, NY, USA) was obtained from Enzo Life Sciences. CIT was dissolved in dimethyl sulfoxide (DMSO; Sigma-Aldrich, St. Louis, MO, USA) to make a stock solution of 20 mM. HepG2 cells were treated with 5 M CIT. Control cells were cultured in medium containing 0.025% DMSO (the concentration of DMSO in 5 M CIT treatment in this study). A separate investigation on the effects of DMSO on the parameters examined in this study showed no significant differences between DMSO-treated and untreated cells.

### 4.2. RNA Interference

To determine the role of autophagy in CIT-induced apoptosis, we used siRNA against Atg5 to block autophagosome formation. HepG2 cells were transfected with either 50 nM siRNA against Atg5 or scrambled control siRNA (Gene Pharma, Suzhou, Jiangsu, China) using transfection reagent Lipofectamine 2000 (Invitrogen, Carlsbad, CA, USA) according to the manufacturer’s instructions. Transfection efficiency was assessed by Westernblot 48 h post transfection [29]. 

### 4.3. Western Blot Analysis

At the end of the designated treatments, the cells were washed twice with ice-cold PBS and completely lysed in the lysis buffer provided with a protein extraction kit (Keygen Biotech, Nanjing, Jiangsu, China). The cell lysate was centrifuged at 16000× *g* and 4 °C for 5 min, and the supernatants containing the total protein were isolated. To assess subcellular relocalization of cathepsin D, Western blot was conducted using cytosolic extracts prepared as previously described [30]. The concentration of total protein was quantified using the BCA method. SDS-polyacrylamide gel electrophoresis was performed, and the proteins were then transferred onto a nitrocellulose membrane. After blocking with 10% non-fat milk, the blots were incubated with primary antibodies against LC3B (Sigma, St. Louis, MO, USA), cathepsin D (Proteintech, Wuhan, Hubei, China), Atg 5 (Cell Signaling Technology, Danvers, MA, USA), or the internal control β-actin (Santa Cruz Biotechnology, Santa Cruz, CA, USA). The blots were then incubated with the appropriate anti-species horseradish peroxidase (HRP)-conjugated secondary antibodies and detected using the SuperSignal West Pico Kit (Thermo Scientific, Rochford, IL, USA) according to the manufacturer’s instructions. The expected protein bands were detected using the Bio-Rad ChemiDoc™ MP imaging system (Bio-Rad Laboraturies, Hercules, CA, USA). The relative abundance of target protein (normalized to β-actin) was measured with the Gel-Pro Analyzer 4.0 software (Media Cybernetics, Rockville, MD, USA, 2001) [31].

### 4.4. Measurement of Lysosomal Membrane Stability by AO Staining

Lysosomal stability was assessed by the AO-relocation method. As a lysosomotropic weak base, AO accumulates in the acidic lysosomal compartment due to proton trapping. AO is also a metachromatic fluorescent dye and its fluorescence emission is concentration dependent. AO exhibits red fluorescence at high concentrations (in intact lysosomes), but green fluorescence at low concentrations (when lysosomal contents diffuse into the cytosol) [13]. In this study, HepG2 cells were seeded out on coverslips in 24-well plates. The cells were treated with 5 µM CIT for 6 h, 12 h, or 24 h. Before incubation with 5 µM CIT for 12 h, the cells were pretreated with 50 nM siRNA against Atg5 for 48 h. The cells were then stained with 1 µg/ml AO (Amresco, Solon, OH, USA) at 37 °C for 15 min, rinsed twice with ice-cold PBS [32]. Samples were observed under a fluorescence microscope (Olympus BX63, OLYMPUS, Shinjuku-ku, Tokyo, Japan). Lysosomal stability was assessed by red AO-fluorescence, using Image-Pro Plus 6.0 software (Media Cybernetics, Rockville, MD, USA, 2006). Fluorescence intensity was expressed in arbitrary units: average pixel value per lysosome or the lysosome area [33].

### 4.5. Measurement of ΔΨm

ΔΨm of CIT-treated HepG2 cells was evaluated by JC-1 staining. Fluorescent dye JC-1 exhibits potential dependent accumulation in mitochondria by a fluorescence emission shift from green (monomeric form: indicating depolarized/low ΔΨm) to red (aggregated form: indicating polarized/normal ΔΨm). In this study, HepG2 cells were seeded out on coverslips in 24-well plates. The cells were treated with 5 M CIT for 6 h, 12 h, or 24 h. Before incubation with 5 M CIT for 12 h, the cells were pretreated with 50 nM siRNA against Atg5 for 48 h. The cells were then incubated with 5 g/mL JC-1 (Beyotime Institute of Biotechnology, Haimen, Jiangsu, China) for 20 min at 37 °C, and washed three times with ice-cold PBS. The viable cells were analyzed using a fluorescence microscope (Olympus BX63, OLYMPUS, Shinjuku-ku, Tokyo, Japan) with a standard FITC filter set for detection of JC-1 monomers and a Cy3 filter set for detection of JC-1 aggregates. The fluorescence intensity was quantified by Image-Pro Plus 6.0 software (Media Cybernetics, Rockville, MD, USA, 2006). ΔΨm was represented by the ratio of red/green fluorescence intensity [34].

### 4.6. Caspase-3 Assay

Caspase-3 activity in cell lysates was determined using the Caspase-3 Colorimetric Assay Kit (Keygen Biotech, Nanjing, Jiangsu, China). HepG2 cells were treated with 5 µM CIT for 6 h, 12 h, or 24 h. Before incubation with 5 µM CIT for 24 h, the cells were pretreated with 50 nM siRNA against Atg5 for 48 h or with 40 µM pepstatin A (Amresco, Solon, OH, USA), a cathepsin D inhibitor, for 4 h, respectively. The cells were lysed by incubating on ice for 20 minutes in ice-cold lysis buffer, and were then centrifuged at 10,000×g for 2 min. Caspase-3 activity in the supernatant was assayed according to the manufacturer’s directions. Caspase-3 activity was expressed as a percentage of control activity [35]. 

### 4.7. Statistical Analysis

Data were expressed as the means ± SD from at least three independent experiments performed in triplicate and analyzed using the SPSS 13.0 statistical software (IBM SPSS Software, Chicago, IL, USA, 2005). The comparisons between groups were analyzed using one-way ANOVA followed by Student-Newman-Keuls (SNK) test, and *p* < 0.05 was considered statistically significant.

## 5. Conclusions

The data demonstrated that CIT induced autophagy-dependent apoptosis in HepG2 cells. CIT probably activated autophagic flux and induced LMP subsequently. CIT-induced LMP caused lysosomal release of cathepsin D, induced MOMP, and triggered mitochondrial apoptosis eventually.
